# A Closer Look at Parental Narratives: A Qualitative Analysis of Parental Entries in Neonatal Research Diaries of Preterm Infants Participating in the REPORT-BPD Feasibility Study

**DOI:** 10.3390/children12081059

**Published:** 2025-08-12

**Authors:** Wisam Muhsen, Ana Guillot Lozano, Jos M. Latour

**Affiliations:** 1 Faculty of Health, University of Plymouth, Plymouth PL6 8BU, UK; ana.guillotlozano@students.plymouth.ac.uk (A.G.L.); jos.latour@plymouth.ac.uk (J.M.L.); 2 Department of Nursing, Zhongshan Hospital, Fudan University, Shanghai 200032, China; 3 Curtin School of Nursing, Curtin University, Perth, WA 6102, Australia

**Keywords:** qualitative analysis, neonatal research diaries, bronchopulmonary dysplasia, echocardiography, preterm infants

## Abstract

**Highlights:**

**What are the main findings?**

- Parental diary entries captured meaningful real-time observations of preterm infants’ health during the first 10 days of life in the NICU.
- These entries showed emotional depth and parental vigilance but lacked the clinical specificity to support prediction of BPD.

**What is the implication of the main finding?**

- Parental observations, while insightful, do not appear to complement echocardiographic data in predicting early BPD development.
- Neonatal diaries may still hold value as a communication tool to enhance family-centred care and parent–staff collaboration in the NICU.

**Abstract:**

**Background/Objectives:** Bronchopulmonary dysplasia (BPD) is a chronic lung disease affecting preterm infants, often resulting in prolonged neonatal intensive care unit (NICU) stays and significant parental stress. The experiences of parents navigating their preterm infant’s early NICU journey are important to support clinical trials to improve infant outcomes. **Aim:** The aim of this study was to explore parental perceptions of their infant’s health progression during the first 10 days of life through personal diary entries and their correlation with the echo scans assessments, as part of the Exploring Right vEntricular function applicability in a Prediction mOdel to identify pReterm infanTs with early BronchoPulmonary Dysplasia (REPORT-BPD) feasibility study. **Methods:** An embedded qualitative design was employed, utilising thematic analysis of 17 parent diaries. Parents of preterm infants (<32 weeks of gestation) admitted to a NICU documented their daily experiences. Thematic analysis was applied to ensure a rigorous, inductive examination of emerging themes. **Findings:** Four main themes were identified: (1) developing parent–infant relationships, highlighting the emotional impact of separation and the significance of bonding; (2) health and well-being of premature infants and family, reflecting parental vigilance, cautious optimism, and emotional distress; (3) parents navigating support and the NICU environment, describing challenges related to medical procedures, communication with staff, and adapting to a highly technical setting; and (4) emotions and protective gestures, illustrating parental resilience, coping mechanisms, and the innate drive to protect their child. **Conclusions:** Parental experiences in the NICU were shaped by emotional turmoil, uncertainty, and the need for support in navigating their infant’s care. Diaries provided an effective means for parents to express their experiences; they could serve as a communication tool in clinical trials to provide a deeper understanding of the development of the recruited preterm infants.

## 1. Introduction

Bronchopulmonary dysplasia (BPD) is a chronic lung disease that affects infants born before 32 weeks of gestation [1]. Diagnosis and assessment of severity cannot take place until 36 weeks post-menstrual age (PMA) or at discharge, when discharge from the neonatal intensive care unit (NICU) is planned earlier [2]. BPD affects preterm infants with higher oxygen requirements due to their premature lungs, and it is one of the most common morbidities of prematurity. BPD leads to extended hospital stays and long-term pulmonary and neurodevelopmental complications, together with high healthcare costs [3,4].

According to the National Neonatal Audit Programme in the United Kingdom (UK), the incidence of moderate and severe forms of BPD is almost a third of all infants born before 32 weeks of gestation [5]. The incidence continues to remain high as the population at risk increases due to improvements in obstetric and neonatal practices, leading to the viability threshold reducing to 22 weeks of gestation, which results in increased survival of at-risk extremely preterm infants [3].

A vascular theory that could explain BPD is the vascular pathogenesis hypothesis. This theory emphasises the intricate interdependence between the developing cardiovascular and respiratory systems in premature infants, suggesting that disruptions in pulmonary vascular development can directly impair alveolar growth and lung maturation [6]. Development of BPD would affect the interlinked capillary bed, which would cause pulmonary hypertension and affect the right ventricle function [7]. The central premise of the REPORT-BPD study is that hemodynamic changes precede the overt deterioration in respiratory function and the eventual development of bronchopulmonary dysplasia (BPD), and this temporal sequence forms the foundation of the study’s predictive model for BPD [8]. The REPORT-BPD feasibility study aims to determine whether a prediction model can be developed from cardiac echo parameters that show right heart strain, indicating pulmonary hypertension, and consequently BPD [9].

Preterm infants with BPD spend a long time as inpatients in the NICU and are more prone to re-hospitalisations [10]. An admission of an infant to the NICU can be stressful and emotional for their parents [11]. The hospital environment is unnatural to many patients due to the technical equipment, noise, medical language, and level of activity [11]. Combining this with the stress of a sick infant worsens the circumstances. It is an unpredictable, often life-threatening, time that can cause post-NICU unresolved issues [12]. Removing the stress of parents whose preterm infant is admitted to the NICU is unattainable but the goal to support them to make the experience more amenable is realistic.

Parental involvement in various activities within the neonatal unit, including participation in clinical research, has been shown to yield significant benefits [13,14]. Parents who have experienced having a preterm infant in neonatal care often possess valuable insights that can inform multiple stages of neonatal research. Their perspectives can contribute to defining research priorities, shaping study protocols, identifying relevant outcome measures, and navigating ethical approval processes. Additionally, parents can play a crucial role in developing patient information leaflets and consent forms, thereby enhancing the clarity and accessibility of these materials for future participants [13,14].

The use of diaries is common in intensive care settings. They allow parents to express their thoughts and for healthcare professionals to update and react to the concerns that parents write in their diaries. Diaries can help in the recognition of parents of an infant who has a prolonged NICU stay who are exhibiting any signs of psychological trauma, so the neonatal team can provide the appropriate psychological support to augment self-preservation and maintain the human connection within the technical environment of the neonatal unit [15]. Given the wide range of benefits that neonatal unit diaries can offer in enhancing practices within neonatal care settings, their use can be valuable in shaping clinical research that actively involves parents and service users. These diaries serve as a meaningful tool for capturing lived experiences, fostering collaboration, and ensuring that research is more patient- and family-centred [13].

This study aimed to explore the perceptions of parents regarding their infant’s health progression in the first 10 days of postnatal life and the parental experiences during this period using individualised neonatal diaries. Additionally, the study sought to examine whether diary entries correlated with clinical findings, such as echocardiographic assessments [9]. This qualitative study was embedded in a mixed-methods observational cohort feasibility study, the REPORT-BPD study. The overall aim of the REPORT-BPD feasibility study was to explore the feasibility of producing a prediction model for the development of bronchopulmonary dysplasia (BPD) using right ventricular function and other parameters as per echo scans in the first 10 days of life. Hence, researchers invited all parents, whose preterm infants were recruited, to document any observed clinical changes related to, e.g., respiratory function, to explore whether parents’ real-time, observational insights could complement and potentially enhance the predictability of echocardiographic parameters for BPD risk. The focus was on whether parental observations might align with early physiological markers of the evolving BPD.

## 2. Materials and Methods

This qualitative study, using personal diaries of parents, is part of a larger observational cohort study. The researchers chose an embedded qualitative study design to explore the contextual and unique nature of parental experiences during the first 10 days of life in the NICU. The Consolidated Criteria for Reporting Qualitative Research (COREQ) guidelines have been used to report the study [16].

### 2.1. Ethical Considerations

All potential participants received both verbal and written information about the study via a participant information sheet (PIS). Participation was voluntary, and confidentiality was assured, with all collected data kept strictly confidential. Participants were informed of their right to withdraw from the study at any time, without being required to provide a reason, and without any impact on their ongoing care or treatment. Following this, those who agreed to participate provided their written consent by signing the informed consent form (ICF).

Medical practitioners who acted as medical delegates in consenting participants were duly authorised, completed Good Clinical Practice training, were experienced clinicians, and were competent to participate according to the ethically approved protocol, principles of GCP, and Declaration of Helsinki. To ensure this, a delegation log was maintained.

The researchers obtained the approval and review of the REPORT-BPD feasibility study, the informed consent sheets, and participant information sheet from the following relevant ethical committees: Health Research Authority, Southwest-Central Bristol Research Ethics Committee (reference: 22/SW/0023, 16 March 2022), and the Faculty of Health Research Ethics & Integrity Committee of the University of Plymouth (reference: 3371, 28 April 2022).

### 2.2. Participants and Settings

The recruitment period was set to be over 18 months, but the targeted sample size of 40 preterm infants was achieved within 11 months, from 1 June 2022 to 5 May 2023. The study included infants born before 32 weeks’ gestation who were admitted to the UHP NICU during this period. A detailed set of inclusion and exclusion criteria was specified in the REPORT-BPD research project protocol [9].

The REPORT-BPD study obtained informed consent from the parents or legal guardians of a total of 40 preterm infants. Each family was provided with a single neonatal research diary. At the end of the data collection period, only 17 completed diaries were available and included in the data analysis. The remaining families’ diaries were empty. Their reason for not engaging with the diaries was not collected. The appearing reasons are the busy NICU and stormy course.

### 2.3. Data Collection

The data collection method was through the neonatal research diaries. Diaries reduce the effect of setting on participants’ reluctance to talk or the influence of non-participants due to increased privacy of the diary; it allows participants to keep it hidden when other people are around. Contrastingly, written data do not convey the same amount of detail about the emotional weight of comments or body language as audio or video recordings would. Nevertheless, diaries allow parents to write about their experiences as they are happening.

Parents of the participating preterm infants were advised to write as much or as little as they wanted and given a leaflet. The leaflet described “how to write a NICU diary” as a guide on what to write, including reflections, feelings, and comments about their child’s health and progress. The diary included 50 blank pages. No limitations on the frequency of note-taking were applied. Copies of the diary were obtained after the 10th DAB for analysis, leaving original copies for their keepsake, and were then transcribed into Word documents by W.M.

### 2.4. Data Analysis

Thematic analysis is a method to identify, analyse, and report patterns within qualitative data. The six steps of thematic analysis were followed, forming a systematic method of data analysis that allows flexibility to fit the research question and data, making it the most suitable method for this question [17].

An inductive data analysis was performed by W.M. and A.G.L., with the support of J.M.L. during discussion and clarification of disagreements, following the six steps for thematic analysis proposed in the design [17]. In the first step, W.M. and A.G.L. familiarised themselves with the text by reading the transcripts. In the second step, both researchers independently coded the text, using NVIVO software (Version 14), and a discussion followed to align the coding in the third step. They reformulated individual codes and potential themes and subthemes. As part of this step, they discussed their agreement and consistency checks. In step four, they scrutinised the themes to generate an accurate thematic map and examined whether the map reflected the codes from the dataset. Then, in step five, the final themes and subthemes were defined, and the last step included the report [17]. This process involved input from J.M.L. during several discussions and the scrutiny of codes, subthemes, and themes.

Also, as part of the qualitative analysis, the researcher examined parental diary entries for descriptions of clinical changes in their preterm infants—such as breathing difficulties, increased respiratory support, or other respiratory-related events—during the first 10 days of life that could be plausibly linked to early signs of bronchopulmonary dysplasia (BPD). These entries were reviewed to explore whether parental observations corresponded with early echocardiographic indicators of BPD risk. The aim was to assess whether the reported clinical signs were sufficiently detailed and closely aligned in time with objective findings to complement echocardiographic data in the early prediction of BPD, potentially.

## 3. Results

The parents of all 40 recruited preterm infants were given a neonatal research diary, with one diary provided per infant. However, only 17 diaries were completed by parents of the recruited preterm infants, comprising both those diagnosed with BPD and those without the condition (Table 1).

The thematic analysis of the filled 17 diaries derived 162 codes, which, after discussion and merging, were summarised into 49 codes. The codes were grouped into 10 subthemes and further categorised into four themes (Table 2). The four themes are (1) developing parent–infant relationships, highlighting the emotional impact of separation and the significance of bonding; (2) health and well-being of premature infants and family, reflecting parental vigilance, cautious optimism, and emotional distress; (3) parents navigating support and the NICU environment, describing challenges related to medical procedures, communication with staff, and adapting to a highly technical setting; and (4) emotions and protective gestures, illustrating parental resilience, coping mechanisms, and the innate drive to protect their child.

The level of completion of the neonatal research diaries by parents of the recruited preterm infants varied significantly. Some entries were very detailed, while others contained only minimal information. This variation is evident in the coding data: five diaries produced fewer than 15 codes, four diaries yielded more than 90 codes, and the remaining eight fell somewhere between 15 and 90 codes.

### 3.1. Theme: Developing Parent–Infant Relationships

This theme highlights the emotional impact of separation and the significance of bonding. Nearly all parents shared insights into their experience in building a relationship with their infant.

#### 3.1.1. Subtheme: The Effects of Familial Separation

The health condition of preterm infants and their extended stay in the NICU led to separations from the infant, between parents and from other children. This separation impacted the emotional well-being of the parents by adding stress, amplifying concerns about the infant’s health while also diminishing the mother’s support network and the security that comes with it: *“Today was a hard day for all 4 of us as we got split up last night, and it feels like a nightmare that never ends. Everywhere I look, all I see is a place where I’ve been with my husband, and the spot where the other twin’s crib used to be is painful to look at.” (D04).*

#### 3.1.2. Subtheme: Building a Relationship with Their Infant

When parents interact with their infants, it positively influences the family’s emotional state. From noticing small behaviour changes and getting to know their infant, through deeply enjoying moments of physical contact, to statements of deep love for their baby. Physical contact was generally valued, either skin-to-skin or just a touch. This physical contact, even if it was for a brief period, gave them a sense of involvement by soothing their baby. Other valuable interactions included changing clothes or diapers, reading stories, or feeding: *“We are impatiently waiting to start cuddle time this evening, daddy is dying to hold his little baby (heart emoji)” (D03).*

These interactions fed into the parents’ sense of familiarity with their child. They expressed this through statements of unconditional love or noticing the infant’s efforts and behavioural changes.

### 3.2. Theme: Health and Well-Being of Premature Infants and Family

When facing their infant’s admission to the NICU, parents often struggled with their own well-being and emotional stability, compounded by uncertainty about what the future might hold. This theme reflects parental vigilance, cautious optimism, and emotional distress

#### 3.2.1. Subtheme: Cautiously Hopeful for a Favourable Outcome

Parents were vigilant of changes in their babies’ parameters, clinical signs, or behaviour.

Negative changes were noticed: *“His oxygen levels went up to 0.51, which was curious as lately our baby went above 0.30, the baby looks like very unsettled and uncomfortable, shivering, wiggling, clinging the fists and moving constantly as is having spasms or cramps.” (D04).*

Contrastingly, some positive changes were recorded too: *“Not much new to report today, which is great–no news is good news!” (D04).*

Also, they often reported balanced positive and negative observations: *“My little angel still looks pretty bruised, but he is getting better.” (D13).*

This range influenced the parents’ state of hope. They balanced focusing on formulating goals, big or small, with having hope for good things and remaining wary when there was positive progress: *“I am told, baby is feisty and doing well, I am very pleased to hear this but still cautious to get too excited” (D33).*

#### 3.2.2. Subtheme: Impact of Infant’s Health on the Family

While parents stayed cautiously hopeful, changes in the infant’s health influenced the family’s well-being. This was in the form of worries, anxiety, and emotional struggles, sometimes leading to sleep disruptions: *“A very hard day for us all! Baby had several big desats throughout the day. Scared the living hell out of us. Are we going to loose our baby–what can we do–help!!” (D30); “My baby was put on monitoring equipment. It was a very overwhelming environment with all the bleeping and monitors” (D24).*

However, other parents described joy: “*When we walked into the room, we were asked if we would like to hold our baby tonight as the baby is behaving quite good. Our hearts immediately exploded with joy and you could be blinded by our smiles that filled the room.” (D03).*

### 3.3. Theme: Parents Navigating the Support and the Environment

The diaries highlighted the significance of the unfamiliar NICU environment. They had to navigate new equipment, terms, schedules, communication systems, and the support available to them, often for the first time. This theme describes challenges related to medical procedures, communication with staff, and adapting to a highly technical setting.

#### 3.3.1. Subtheme: Different Sources of Support

Support was noticed by many parents, including from the hospital staff, each other, and the extended family. The staff provided emotional support by reassuring the parents during anxious times. They provided practical support, such as improving their health and getting engaged with their feeding. The latter further increased the emotional support by completing their need to be involved in their baby’s care and have some control over their child’s care: *“All the staff, every single person, continue to be amazing, caring and supportive. Without that my expressing wouldn’t have gone as well as it has done for our son xx.” (D28).*

Family support was key for navigating the new environment, from the partner, extended family, and even other children. The partner shared the family burden and cared for the mother’s health. As one mother mentioned, *“Dad allowed our baby to get settled in with nursing team whilst dad went to check on mum” (D11)*. Visits from extended family were reassuring and provided a welcome change of environment. As one parent described, *“Today, baby met with their siblings and grandmother which was lovely for baby and all of us” (D24).*

#### 3.3.2. Subtheme: Effects of the Unfamiliar Neonatal Unit Environment and Parental Adaptation

The NICU presented new experiences for parents. Their engagement in caring for the baby was different, as well as feeding their baby. This is something that parents have to adapt to, and these diaries captured some of their thoughts. For example, the mother in D26 said *“I shall assist in feeds again today, it is nice to feel useful and not helpless for my baby.”*

Communication issues influence the adaptation process since they can lead to receiving bad news, leaving parents feeling “shattered”, heartbroken, or guilty. They also have to coordinate care with a bigger team, which comes with adjustments to communication style, the ever-changing ward round schedule, and vocabulary. This leads to worries about missing important updates, chasing staff for updates, or feeling frustrated with schedule changes: *“We completely missed today’s morning handover for our baby, so we were updated by the caring nurse as best as she could. It is extremely frustrating that the handover had started so early, as we are working hard on monitoring a schedule for the babies to make sure we are present for the most important events of the day.” (D03).*

#### 3.3.3. Subtheme: Perception of Different Aspects of Clinical Care and Treatment

This subtheme describes parents navigating clinical concepts. Part of this is noticing the continuous changes in their infant’s health, improvements in clinical signs and behaviours, worrying about them, or unexpected setbacks.

They then try to interpret what it all means, which includes observing oxygen support changes, phototherapy status, feed changes, etc. Inevitably, this leads to having to face their infants receiving care, management, or interventions that they would not receive in other circumstances. They attempt to make the best of all the new terms being used, but still worry: *“Baby was due to have a blood transfusion today which I was worried about this was done through a line and baby was completely unaware and settled during.” (D24).*

#### 3.3.4. Subtheme: Parental Relationships and Communication Between Themselves and Others

Parents constantly communicated with each other and staff to stay updated on their baby’s care, which they needed to digest and interpret. This relieved previous anxieties, helped understand management and adverse effects, and adjusted to the NICU. They also build a rapport and begin to trust them. Contrastingly, mixed opinions, miscommunications, or assumptions sometimes created confusion: *“I believe baby will be fed today so I presume the radiologist have seen and okayed the x-rays of the tummy (although this hasn’t been confirmed to me as yet) fingers crossed, my baby will do well with some food today and not vomit it up like before.” (D27).*

### 3.4. Theme: Emotions and Protective Gestures: Welcoming Our Little One

Being in the NICU is emotionally charged for them and filled with attempts to fulfil their instinctive nature to protect their infants. As they are welcoming their child into the world, they develop coping mechanisms to adjust to the situation. This theme illustrates parental resilience, coping mechanisms, and the innate drive to protect their child.

#### 3.4.1. Subtheme: Parental Protective Instincts—Creating a Shield of Care

Parents expressed their strong desire to protect their infant. This led them to frustrations when impulses could not be met: “*I hate to disturb baby, hate it when baby cries in the incubator, and I cannot comfort baby.” (D33)*. Another example was wanting to protect the baby, “*Almost lifted their own head slightly (a part of me worries, baby is trying to run before can walk)” (D28).*

#### 3.4.2. Subtheme: Parental Coping Mechanisms for What Is Going on Around Them—A Show of Resilience

Parents developed various coping mechanisms, like guarded optimism, enjoying physical contact, or engaging in the infant’s care. The parent from (D33) described allowing herself to begin to have hopes for the future. Another mother used fiction to hope for a better future for her child: *“Mummy got to read to her baby a nice story today, called “the magic Finger”. I wish, I had a superpower and point my finger at my little babies and take all the pain and the premature issues away. I guess that is what all parents would do! I would even for as far as selling my own soul for the price of both of them.” (D03).*

Further representative excerpts from the parental diaries are provided (Table A1, Appendix A).

## 4. Discussion

Developing a relationship between the parents and the infant was key to the parental experience. The change in their parental role, where the caring and protecting responsibilities became shared with the staff, became a source of stress. This was improved by staff support with feeding or skin-to-skin time. Other studies have supported how increasing engagement in the care of their child helps parents cope [11,18,19]. Separation from the child and the entire family exacerbated the emotional toll of this concept [20]. Contrastingly, bonding moments or any interaction facilitated a sense of purpose and connection with their child, which emphasises the importance of prioritising family-centred care [21].

The findings of this study were related to the experiences of parents navigating the NICU journey with the ups and downs in their preterm infants’ clinical condition. The themes identified highlight the multifaceted challenges and coping mechanisms parents employ during this critical period.

Regarding family and infant well-being, a clear theme emerged: parents were often able to recognise changes in their preterm infants’ clinical condition. However, these observations—whether indicating improvement or deterioration—were non-specific and could be attributed to various conditions associated with prematurity. For example, fluctuations in oxygen saturation levels might result from a range of factors, such as infection, stress, pain, or other transient issues [22,23].

While parental observations can provide healthcare professionals with valuable insights into the infant’s current clinical status, they do not have the potential to complement the echocardiogram findings as a predictive tool for BPD.

The highly technical and unfamiliar environment of the NICU becomes a significant challenge for parents to navigate and understand the available support. Studies show that communication with the staff and ongoing information helps build a relationship and control parents’ anxiety [24,25]. In our study, parents highlighted the significance of updates and the frustration of missing ward rounds. This study has also shown how equipment can get in the way, and clinical care or treatments often lead to emotional reactions and hinder parental adaptation. However, other studies have shown a balance between this and positive perceptions of the equipment, such as reassurance provided by being able to visualise vitals in monitors [19].

The admission of a preterm infant to the NICU can be an emotionally draining experience for parents, often accompanied by feelings of guilt, fear, anxiety, or sadness [26]. This study showed that parents would develop coping mechanisms and demonstrate their protective instincts as a response. A systematic review by Obeidat et al. (2009) [27] compiled qualitative research on the experience of parents in the NICU, and [28] published an updated review that looked at this topic exclusively in mothers [27,28]. Similar themes have come to light from other studies referenced, as well as this one. They concluded a need for supportive neonatal interventions to promote positive experiences, with family-centred care (FCC) [21].

Evidence suggests that diaries can serve as a valuable tool in evaluating neonatal interventions, particularly those aimed at enhancing FCC [29,30,31]. Our findings on NICU diaries indicate they serve as a data collection method and can address the concerns parents face related to the objectives of the REPORT-BPD study [9,31]. Remarkably, the themes developed suggest that these diaries could act as a valuable communication tool between parents and staff [32]. For instance, they allow parents during meetings to bring up concerns they have written and help neonatal unit staff update parents when information is missing. Neonatal unit staff, for example, nurses, can read through the entries to ensure any misunderstandings or worries are addressed. Diaries foster a sense of involvement in the infant’s care, improving parents’ navigation of the NICU environment and offering a coping mechanism by reducing their anxieties and ensuring their concerns are heard [33]. Therefore, we recommend introducing them as a means of facilitating bidirectional communication between parents and staff. We suggest further research into the experiences of parents using diaries in the NICU to enhance data collection and improve FCC. This recommendation aligns with previous evidence showing that nursing interventions can lead to positive psychosocial outcomes [31,32].

Furthermore, unlike interviews, which typically elicit structured and often retrospective accounts, neonatal diaries provide a distinctive, real-time record of parents’ emotions, experiences, and observations as they occur throughout the NICU journey [30]. While interviews and focus groups offer rich, in-depth insights and the opportunity for follow-up questioning, they can be subject to limitations such as recall bias or the influence of social desirability [34,35]. In contrast, diaries capture the immediacy and authenticity of parental perspectives at the moment of experience. Research indicates that diaries can uncover nuanced, day-to-day shifts in both the infant’s condition and parental coping strategies—details that may not surface during periodic interviews [31]. This makes them especially valuable for gaining longitudinal understanding and documenting clinically meaningful observations within naturalistic contexts. Ultimately, diaries, interviews, and focus groups are all valid qualitative data collection methods; the choice among them depends on the specific research aims and objectives.

### Limitations

The first impression after an in-depth analysis of the neonatal research diaries indicated differences in the extent of parental completion. This variation in diary completion may reflect differences in how parents engaged with the research process. Factors such as personal coping styles, emotional involvement, time availability, or differing expectations about their role in the study could have influenced the depth and frequency of their entries. The wide range in the number of codes also suggests diverse perspectives on the neonatal care experience—some parents may have viewed the diary as a way to document and process their emotions in detail. In contrast, others may have used it more sparingly or felt less inclined to engage fully. This finding highlights the importance of considering individual differences when interpreting qualitative data from parental diaries. It may also indicate a need for more precise guidance or support to ensure more consistent participation in future diary-based studies involving parents of preterm infants. One potential use of the neonatal research diary is as a communication tool between healthcare professionals and parents. It could serve to document regular updates for parents, for example, overnight when they are not present, allowing them to review the information later in the day after the night team has finished their shift. This approach would transform the diary into a two-way conversation rather than a one-sided record where parents are the only ones writing entries. Such interaction may encourage more consistent participation and deeper engagement from parents in completing the diaries.

As is often seen in studies involving neonatal research diaries, parents who choose to participate are typically already interested in contributing to the research topic. It would therefore be valuable to gather input from other parents who may not have a particular interest in neonatal research beyond providing consent and agreeing to their child’s participation.

The collected neonatal research data covered the first 10 days following birth. As a result, the analysis was limited to diaries completed within this timeframe, as some parents may have started writing their entries after the initial, often stressful, phase of their NICU experience.

## 5. Conclusions

This study adds to the existing literature on parental experiences in the NICU and the use of research diaries as a data collection tool. It offers valuable insights into the emotional and psychological challenges parents face during the first 10 days of their infant’s life in the NICU, shedding light on their feelings of lost control and the coping strategies they develop. However, no significant potential association was observed between parental entries in neonatal diaries and echocardiographic findings as a predictor of BPD. The clinical observations recorded by parents were predominantly general and non-specific, suggesting that they are unlikely to contribute meaningfully to the predictive accuracy of echocardiographic parameters for bronchopulmonary dysplasia (BPD). Despite the study limitations, it highlights the potential of diaries as a means of facilitating communication between families and healthcare providers. Future research should investigate how integrating NICU diaries into routine care could support and enhance FCC practices.

## Figures and Tables

**Table 1:** Patient details whose diaries were included in the study.

No.	Diary No.	Infant’s BPD Outcome (YES/NO)	No.	Diary No.	Infant’s BPD Outcome (YES/NO)
**1**	D02	NO	**10**	D22	YES
**2**	D03	YES	**11**	D24	NO
**3**	D04	YES	**12**	D25	YES
**4**	D11	NO	**13**	D26	YES
**5**	D12	NO	**14**	D27	YES
**6**	D13	YES	**15**	D28	YES
**7**	D17	NO	**16**	D30	YES
**8**	D18	YES	**17**	D33	YES
**9**	D20	YES			

**Table 2:** Themes and subthemes.

Theme	Subtheme
Developing parent–infant relationships	The effects of family separation
	Building a relationship with their infant
Health and well-being of premature infants and family	Cautiously hopeful for a favourable outcome
	Impact of infant’s health on the family
Parents navigating the support and the environment	Different sources of support
	Effect of the unfamiliar neonatal unit environment and parental adaptation
	Perception of different aspects of clinical care and treatment
	Parental relationships and communication between themselves and others
Emotions and protective gestures: welcoming our little one	Parental protective instincts—creating a shield of care
	Parental coping mechanisms for what is going on around them—a show of resilience

## Data Availability

The raw data supporting the conclusions of this article will be made available by the authors on request.

## Abbreviations

The following abbreviations are used in this manuscript:

ANNP	Advanced Neonatal Nurse Practitioner
BPD	Bronchopulmonary Dysplasia
COREQ	Consolidated Criteria for Reporting Qualitative Research
FCC	Family-Centred Care
ICU	Intensive Care Unit
LP	Lumbar Puncture
NICU	Neonatal Intensive Care Unit
NVivo	Qualitative Data Analysis Software
PMA	Post-Menstrual Age
RCT	Randomised Controlled Trial
REPORT-BPD	Exploring Right vEntricular function applicability in a Prediction mOdel to identify pReterm infanTs with early BronchoPulmonary Dysplasia
TLA	Three-Letter Acronym

## Appendix A

**Table A1:** Additional references for themes and subthemes.

Theme	Subtheme	Reference
Developing parent–infant relationships	The effects of familial separation	“We wanted for baby daddy to come as I wanted him to be there, but he couldn’t stay long enough as he had to get back home to pick up baby’s brothers and sister.” (D33) “I can’t seem to stop crying and worrying about my 2 babies were split apart and my husband who is my only support network. I feel crushed and sad without him and I don’t know if I have enough strength to carry on with a fake smile in the morning.” (D04)
	Building a relationship with their infant	“he was very sad-kicking all over and started crying, so I took my baby out to feed on me skin to skin, which calmed my baby down immediately!” (D02) “Our baby showed real determination and the breathing improved.” (D11) “Today is the first time I’ve seen my baby without the cover on baby’s head for jaundice. I love seeing my little baby.” (D27)
Health and well-being of premature infants and family	Cautiously hopeful for a favourable outcome	“All that counts for us right now is that our babies come home with us when they are ready” (D04) “I hope we can get on top of this infection quickly to take some stress of baby’s little body and give baby the chance to fight and deal with what is going on with his lungs.” (D03) “At the same time, I am scared to get my hopes up too much as baby is so tiny and it is early days.” (D33)
	Impact of infant’s health on the family	“Unfortunately, I still felt very shocked and overwhelmed and was sick from the medication so I couldn’t stay long” (D13) “Seeing my baby so small and innocent attached to all those lines and tubes is a feeling that no mother can describe. All you can do is to burst in tears and let emotions and tears sweep you away. I did feel so much love instantly that I thought my heart could crack! My wonderful miracle came into the world- head first!” (D03)
Parents navigating the support and the environment.	Different sources of support	“They’ve also been a huge calming help for a worried, hormonal mummy; they always know the right thing to say.” (D28) “We find it very kind of consultant (Sam) and the caring nurses of the day to have the patience of explaining things over and over again. I am aware that I might the same thing 10 times in a row, but everyone is being very nice and polite and always taking the time to ensure we understand what is happening. It is so comforting!” (D04) “Baby got visits from all grandparents today. Which was lovely.” (D30)
	Effect of the unfamiliar neonatal unit environment and parental adaptation	“Finding morning meetings very essential for us to understand (try our best) the ensemble of the situation.” (D03) “the consultant came to see us to let us know that our little baby has a bleeding on the lungs and it is hard to tell whether baby would make it or not. We stayed by the cot side and comforted our baby as much as a parent can do.” (D04) “just need to get used to the night-time pumps.” (D22)
	Perception of different aspects of clinical care and treatment	“Baby’s LP was done earlier that expected in the day and when we returned to see baby it was just a small plaster on baby’s back which again baby didn’t seem phased by.” (D24) “Big mistake, baby was given too high amount for too long without checking levels. 6 h later blood sugars at 1.7 central-emergency glucose given and fortunately baby responded.” (D30)
	Parental relationships and communication between themselves and others	“ANNP says that is normal as baby may have some secretions, but I remember another ANNP telling us that when that happens baby can test it with the red light to widentify if there is any air trapped.” (D04) “The consultants have also been great at explaining things and so reassuring.” (D13) “We were told they needed to rule out meningitis which would mean a lumbar puncture. This terrified us with possible complications.” (D24)
Emotions and protective gestures: welcoming our little one	Parental protective instincts—creating a shield of care	“The instant desire to protect the baby and keep baby safe was overwhelming” (D30) “It is very hard when baby cries in the incubator, though baby did settle when I put my hands in. it is hard to think baby could be crying in there when I am not there and I wouldn’t know, because I can’t be there 24/7.” (D33)
	Parental coping mechanisms (for what is going on around them)—a show of resilience	“CRAZY day. So grateful to be at the right place at the right time. Was a very emotional day facing the fact that my baby almost didn’t survive, but overall super grateful and proud of our little baby.” D02 “Definitely worries about my baby’s health, but trying to stay positive.” D22 “Baby seemed to have so many tubes attached which was scary to see, but reassuring baby was getting the help.” D24
